# Supplementary material for: Bakta: rapid and standardized annotation of bacterial genomes via alignment-free sequence identification
Source: Microb Genom. 2021 Nov 5;7(11):000685. doi: 10.1099/mgen.0.000685 (PMC8743544; doi:10.1099/mgen.0.000685)
Supplement: Supplementary material 1 [file mgen-7-0685-s001.pdf]

Bakta: Rapid & standardized annotation of bacterial genomes via alignment-free sequence identification

Author names:

Oliver Schwengers[1,*], Lukas Jelonek[1], Marius Dieckmann[1], Sebastian Beyvers[1], Jochen Blom[1] Alexander Goesmann[1]

Affiliation:

[1] Bioinformatics and Systems Biology, Justus Liebig University Giessen, Giessen, 35392, Germany

[*] Corresponding author:

E-mail: oliver.schwengers@cb.jlug.de (OS)

Keywords:

genome annotation, bacteria, plasmids, whole-genome sequencing

URLs:

GitHub: https://github.com/oschwengers/bakta

Zenodo: DOI 10.5281/zenodo.4247252

Web: https://bakta.computational.bio

Supplemental Figures

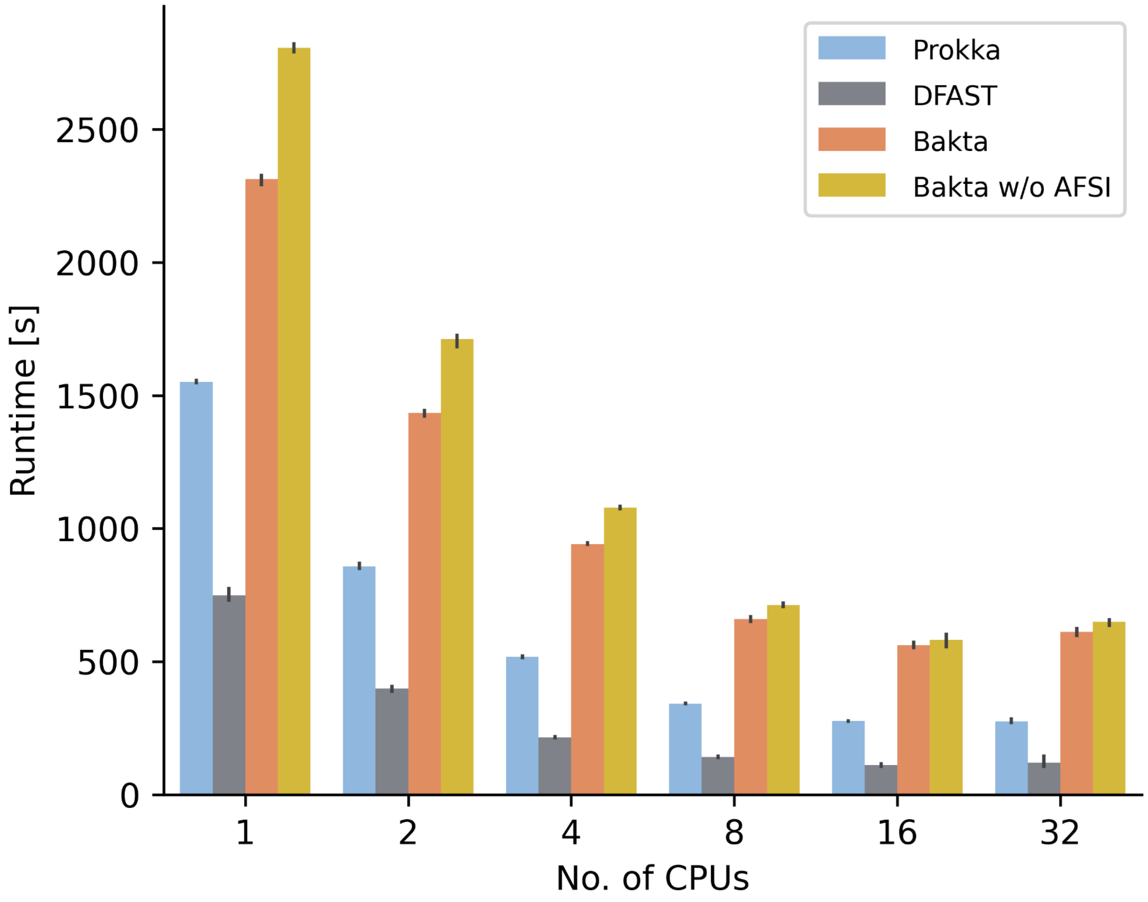

Supplemental Figure S1. Comparison of wall clock runtimes. Runtimes of Prokka, DFAST, Bakta and Bakta w/o AFSI annotating a *Pseudocitrobacter* genome were measured three consecutive times using varying numbers of CPUs on a server machine with 4 Intel Xeon E5-4627 CPUs and 40 cores in total.

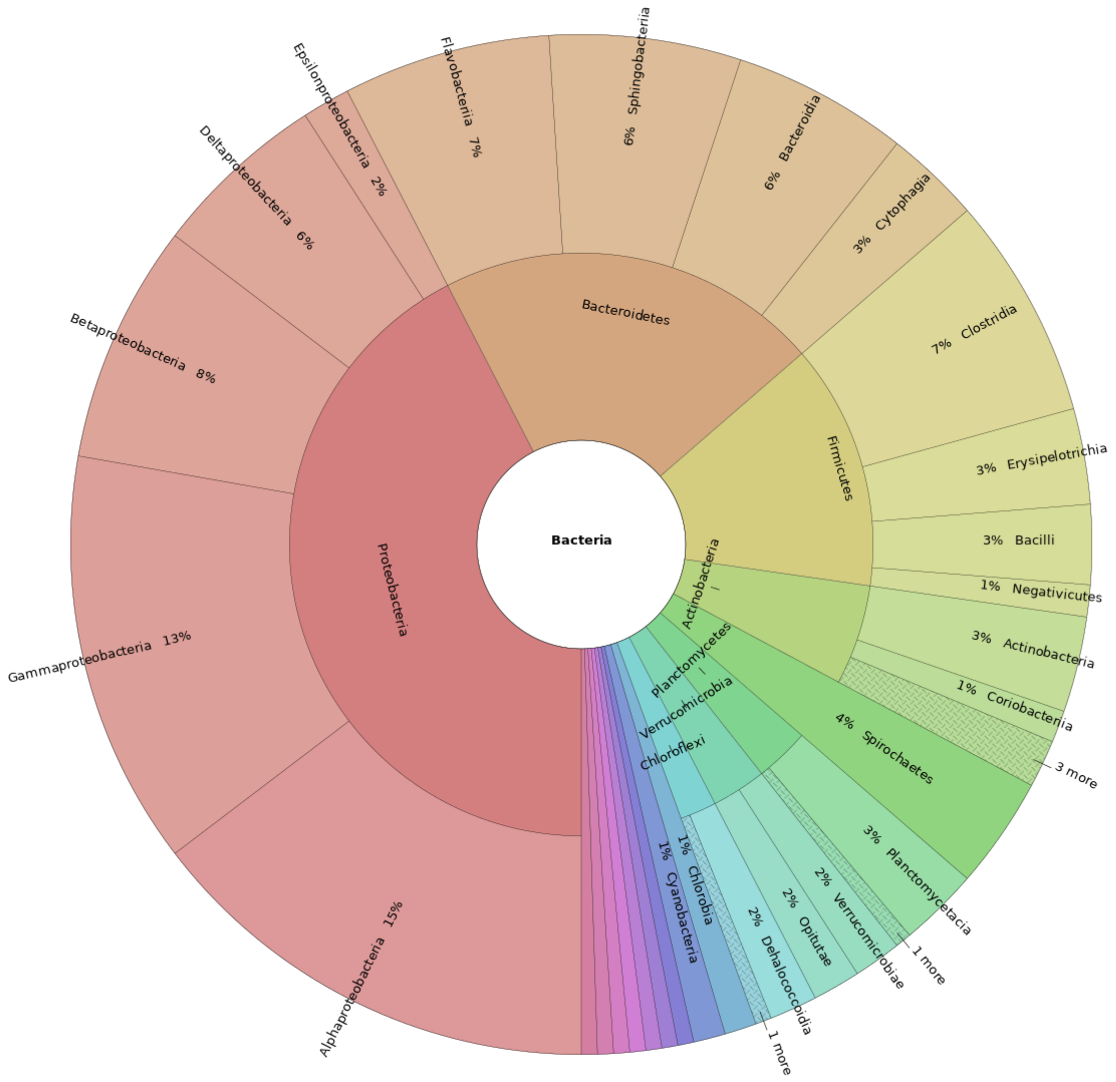

Supplemental Figure S2. Taxonomic distribution of metagenomic-assembled benchmark genomes. 198 bacterial MAGs were selected from 7,903 published MAGs [1] by CheckM [2] complete and contamination scores larger than or equal to 95.0 and smaller than or equal to 1.0, respectively.

Supplemental Tables

Supplemental Table S1. Comparison of functional annotations and identification of coding sequences on 35 selected genomes from RefSeq

Genome / Tool	Total CDS	Hypothetical CDS[a]	Identified CDS[b]	Total sORF[c]
Pseudomonas aeruginosa PAO1 (GCF_000006765.1)				
Prokka	5,671	38.3% (2,172)	-	-
DFAST	5,727	33.5% (1,921)	-	-
Bakta	5,685	5.8% (330)	97.6 % (5,549)	4
Bakta w/o AFSI	5,682	6.5% (372)	-	1
Staphylococcus aureus subsp. aureus NCTC 8325 (GCF_000013425.1)				
Prokka	2,630	20.6% (542)	-	-
DFAST	2,676	17.5% (467)	-	-
Bakta	2,644	4.8% (128)	99.3% (2,626)	13
Bakta w/o AFSI	2,636	6.2% (163)	-	5
Staphylococcus epidermidis ATCC 14990 (GCF_006094375.1)				
Prokka	2,259	20.6% (465)	-	-
DFAST	2,294	19.7% (458)	-	-
Bakta	2,264	7.4% (167)	97.2% (2,200)	5
Bakta w/o AFSI	2,263	8.1% (184)	-	4
Enterococcus faecium ATCC 8459 = NRRL B-2354 (GCF_000336405.1)				
Prokka	2,724	22.5% (613)	-	-
DFAST	2,759	26.8% (740)	-	-
Bakta	2,726	10.9% (298)	96.7% (2,637)	2
Bakta w/o AFSI	2,724	11.9% (325)	-	0

Klebsiella pneumoniae ATCC BAA-2146 (GCF_000364385.3)				
Prokka	5,510	28.7% (1,584)	-	-
DFAST	5,606	21.6% (1,211)	-	-
Bakta	5,528	5.9% (328)	98.6% (5,451)	17
Bakta w/o AFSI	5,519	6.6% (363)	-	10
Listeria monocytogenes R2-502 (GCF_000438585.1)				
Prokka	3,028	38.6% (1,168)	-	-
DFAST	3,054	24.8% (758)	-	-
Bakta	3,036	7.2% (218)	97.5% (2,959)	5
Bakta w/o AFSI	3,035	8.0% (243)	-	4
Escherichia coli O157:H7 Sakai (GCF_000008865.2)				
Prokka	5,374	29.7% (1,598)	-	-
DFAST	5,337	23.0% (1,229)	-	-
Bakta	5,449	4.0% (216)	99.8% (5,436)	76
Bakta w/o AFSI	5,404	4.8% (261)	-	32
Enterobacter cloacae GGT036 (GCF_000770155.1)				
Prokka	4,479	24.3% (1,089)	-	-
DFAST	4,496	15.5% (695)	-	-
Bakta	4,491	4.3% (192)	95.5% (4,287)	11
Bakta w/o AFSI	4,488	4.7% (210)	-	8
Salmonella enterica subsp. *enterica* serovar Infantis 1326/28 (GCF_000953495.1)				
Prokka	4,360	23.7% (1,035)	-	-
DFAST	NA	NA (NA)	-	-
Bakta	4,375	2.9% (126)	97.0% (4,244)	16
Bakta w/o AFSI	4,366	3.3% (146)	-	8
Acinetobacter baumannii ab736 (GCF_002116925.1)				
Prokka	3,712	43.3% (1,609)	-	-
DFAST	3,711	28.0% (1,039)	-	-

Bakta	3,713	13.3% (493)	99.9% (3,710)	1
Bakta w/o AFSI	3,713	14.8% (548)	-	1
Bacillus subtilis subsp. *subtilis* 168 (GCF_000009045.1)				
Prokka	4,214	32.2% (1,358)	-	-
DFAST	4,242	26.4% (1,119)	-	-
Bakta	4,225	1.7% (72)	97.8% (4,130)	8
Bakta w/o AFSI	4,221	2.2% (91)	-	6
Serratia marcescens subsp. *marcescens* ATCC 13880 substr. Sm_S95_jyu2015 (GCF_017299435.1)				
Prokka	4,734	28.0% (1,324)	-	-
DFAST	4,814	20.4% (983)	-	-
Bakta	4,746	5.4% (257)	96.7% (4,590)	10
Bakta w/o AFSI	4,743	6.1% (291)	-	7
Vibrio cholerae MS6 (GCF_000829215.1)				
Prokka	3,550	35.7% (1,267)	-	-
DFAST	3,549	17.6% (623)	-	-
Bakta	3,556	5.8% (205)	97.6% (3,469)	4
Bakta w/o AFSI	3,553	6.3% (225)	-	2
Vibrio parahaemolyticus VPD14 (GCF_004006515.1)				
Prokka	4,561	40.1% (1,831)	-	-
DFAST	4,549	19.0% (865)	-	-
Bakta	4,567	9.9% (452)	95.5% (4,362)	4
Bakta w/o AFSI	4,567	10.3% (471)	-	4
Borreliella burgdorferi B31_NRZ (GCF_002151505.1)				
Prokka	1,339	63.1% (845)	-	-
DFAST	1,352	44.4% (600)	-	-
Bakta	1,339	12.4% (166)	91.6% (1,226)	0
Bakta w/o AFSI	1,339	14.1% (189)	-	0

Campylobacter jejuni subsp. *jejuni* NCTC 11168 (GCF_000009085.1)				
Prokka	1,658	37.9% (629)	-	-
DFAST	1,662	19.3% (321)	-	-
Bakta	1,660	5.5% (91)	95.1% (1,579)	2
Bakta w/o AFSI	1,659	6.8% (112)	-	1
Clostridioides difficile BR81 (GCF_002007885.1)				
Prokka	3,592	41.7% (1,498)	-	-
DFAST	3,649	20.3% (741)	-	-
Bakta	3,596	6.1% (220)	99.6% (3,581)	4
Bakta w/o AFSI	3,593	7.3% (264)	-	1
Clostridium botulinum DFPST0029 (GCF_003058345.1)				
Prokka	3,475	43.1% (1,499)	-	-
DFAST	3,521	24.6% (866)	-	-
Bakta	3,478	8.9% (309)	96.6% (3,359)	2
Bakta w/o AFSI	3,476	10.2% (354)	-	1
Clostridium tetani 12124569 (GCF_000967115.2)				
Prokka	2,849	46.9% (1,335)	-	-
DFAST	2,874	37.7% (1,084)	-	-
Bakta	2,851	15.5% (441)	84.2% (2,401)	0
Bakta w/o AFSI	2,851	16.3% (464)	-	0

Streptococcus pneumoniae NT_110_58 (GCF_000817005.1)				
Prokka	2,246	45.3% (1,017)	-	-
DFAST	2,254	31.3% (706)	-	-
Bakta	2,267	7.1% (162)	99.7% (2,258)	20
Bakta w/o AFSI	2,265	9.6% (217)	-	17
Streptococcus pyogenes NGAS638 (GCF_001267845.1)				
Prokka	1,699	37.3% (634)	-	-

DFAST	1,687	34.3% (578)	-	-
Bakta	1,703	5.3% (91)	95.3% (1,624)	4
Bakta w/o AFSI	1,701	6.5% (110)	-	2
Corynebacterium diphtheriae NCTC11397 (GCF_001457455.1)				
Prokka	2,328	45.4% (1,058)	-	-
DFAST	2,316	37.4% (867)	-	-
Bakta	2,328	14.5% (338)	99.9% (2,326)	0
Bakta w/o AFSI	2,328	15.0% (350)	-	0
Treponema pallidum subsp. *pallidum* X-4 (GCF_005885795.1)				
Prokka	978	45.9% (449)	-	-
DFAST	982	38.7% (380)	-	-
Bakta	978	6.2% (61)	77.2% (755)	0
Bakta w/o AFSI	978	7.2% (70)	-	0
Helicobacter pylori Puno135 (GCF_000224555.1)				
Prokka	1,537	45.1% (693)	-	-
DFAST	1,538	28.0% (430)	-	-
Bakta	1,541	11.2% (173)	92.9% (1,431)	4
Bakta w/o AFSI	1,540	12.0% (185)	-	3
Rickettsia rickettsii Iowa (GCF_000017445.4)				
Prokka	1,427	56.6% (808)	-	-
DFAST	1,365	53.0% (723)	-	-
Bakta	1,428	28.4% (406)	87.5% (1,249)	0
Bakta w/o AFSI	1,428	29.6% (422)	-	0
Legionella pneumophila L10-023 (GCF_001610735.1)				
Prokka	3,533	52.3% (1,848)	-	-
DFAST	3,471	36.3% (1,261)	-	-
Bakta	3,352	17.8% (628)	92.0% (3,085)	0
Bakta w/o AFSI	3,532	18.7% (660)	-	0

Yersinia enterocolitica FORC_002 (GCF_000987925.1)				
Prokka	4,327	30.3% (1,313)	-	-
DFAST	4,306	20.2% (868)	-	-
Bakta	4,338	7.6% (328)	93.8% (4,068)	8
Bakta w/o AFSI	4,336	7.8% (338)	-	6
Bacillus thuringiensis ATCC 10792 (GCF_002119445.1)				
Prokka	6,327	50.8% (3,211)	-	-
DFAST	6,388	36.2% (2,315)	-	-
Bakta	6,341	12.7% (807)	98.9% (6,271)	8
Bakta w/o AFSI	6,339	14.7% (931)	-	5
Lactobacillus casei DSM 20011 (GCF_000829055.1)				
Prokka	2,890	44.6% (1,288)	-	-
DFAST	2,905	34.1% (990)	-	-
Bakta	2,893	13.7% (397)	92.7% (2,681)	0
Bakta w/o AFSI	2,891	14.7% (425)	-	0
Streptomyces antibioticus DSM 41481 (GCF_012044595.1)				
Prokka	7,612	55.6% (4,231)	-	-
DFAST	7,831	41.3% (3,233)	-	-
Bakta	7,620	16.7% (1,276)	82.5% (6,287)	1
Bakta w/o AFSI	7,619	17.5% (1,335)	-	0
Corynebacterium jeikeium NCTC11914 (GCF_900477975.1)				
Prokka	2,057	40.7% (838)	-	-
DFAST	2,078	34.0% (707)	-	-
Bakta	2,060	8.3% (170)	99.8% (2,055)	2
Bakta w/o AFSI	2,059	10.2% (209)	-	1
Xanthomonas campestris M28 (GCF_014841015.1)				
Prokka	4,326	46.7% (2,022)	-	-
DFAST	4,358	29.2% (1,274)	-	-

Bakta	4,333	12.9% (559)	84.6% (3,664)	2
Bakta w/o AFSI	4,332	13.9% (600)	-	1
Sinorhizobium meliloti SM11 (GCF_000218265.1)				
Prokka	6,913	47.7% (3,298)	-	-
DFAST	6,927	29.2% (2,025)	-	-
Bakta	6,920	14.4% (998)	94.6% (6,547)	2
Bakta w/o AFSI	6,920	15.3% (1,058)	-	2
Sorangium cellulosum So ce 56 (GCF_000067165.1)				
Prokka	9,627	65.4% (6,297)	-	-
DFAST	9,933	70.6% (7,012)	-	-
Bakta	9,654	25.6% (2,473)	86.2% (8,326)	0
Bakta w/o AFSI	9,654	26.4% (2,549)	-	0
Thiobacillus denitrificans ATCC 25259 (GCF_000012745.1)				
Prokka	2,814	42.3% (1,190)	-	-
DFAST	2,842	36.6% (1,039)	-	-
Bakta	2,818	11.6% (326)	95.2% (2,682)	0
Bakta w/o AFSI	2,818	11.7% (330)	-	0
**Total**				
Prokka	130,360	41.2% (53,656)	-	-
DFAST	127,053	31.6% (40,128)	-	-
Bakta	130,683	10.6% (13,902)	94.2% (123,105)	235
Bakta w/o AFSI	130,573	11.5% (15,066)	-	132

Note: Prokka, DFAST and Bakta were executed with default parameters providing the following information relevant to the annotation workflow: genus, species and assembly status. A detailed list of command lines is provided in Supplemental Notes S3; Bakta w/o AFSI: Bakta without alignment-free sequence identification

[a] Protein sequences of unknown function, *i.e.* product denoted as either *hypothetical protein*, *putative protein, uncharacterized protein* or *conserved predicted protein*.

[b] CDS identified with assigned public database identifiers from either RefSeq, UniParc or UniRef.

[c] Short ORFs shorter than 29 amino acids

Supplemental Table S2. Functional annotation and protein identification benchmark set comprising 362 selected genomes from GenBank

**GenBank accessions**
GCA_018141845.1, GCA_016865485.1, GCA_016865505.1, GCA_018228645.1, GCA_018228685.1,
GCA_019203805.1, GCA_902502875.2, GCA_014191545.1, GCA_018127705.1, GCA_009497875.2,
GCA_013467635.1, GCA_013467655.1, GCA_015476115.1, GCA_017948305.1, GCA_018672315.1,
GCA_017798125.1, GCA_018866245.1, GCA_015243575.1, GCA_016458825.1, GCA_017569285.1,
GCA_018118585.1, GCA_018128945.1, GCA_016865015.1, GCA_016865035.1, GCA_017134815.1,
GCA_015693905.1, GCA_016887485.1, GCA_016350165.1, GCA_016887505.1, GCA_003352025.1,
GCA_018394295.1, GCA_018394315.1, GCA_018622495.1, GCA_018622675.1, GCA_018069705.1,
GCA_018398915.1, GCA_004376055.2, GCA_017798145.1, GCA_017309585.1, GCA_015482585.1,
GCA_014218215.1, GCA_013457415.1, GCA_017161075.1, GCA_017945865.1, GCA_017751145.1,
GCA_016806125.1, GCA_010681825.2, GCA_019355395.1, GCA_014023125.1, GCA_015099395.1,
GCA_014873095.1, GCA_013745115.1, GCA_016865445.1, GCA_017357865.1, GCA_013874835.1,
GCA_014041935.1, GCA_014337215.1, GCA_014337195.1, GCA_014337155.1, GCA_014337175.1,
GCA_016126655.1, GCA_016591975.1, GCA_015679245.1, GCA_017808555.1, GCA_017569265.1,
GCA_017569305.1, GCA_016894345.1, GCA_016925615.1, GCA_016916535.1, GCA_017498545.1,
GCA_017569225.1, GCA_018437505.1, GCA_017945845.1, GCA_907165195.1, GCA_019285715.1,
GCA_018604165.1, GCA_019195455.1, GCA_018916745.1, GCA_014654535.1, GCA_000177275.2,
GCA_000463875.1, GCA_000419805.1, GCA_010206225.2, GCA_000494665.1, GCA_000494645.1,
GCA_001657615.1, GCA_001644275.1, GCA_001644325.1, GCA_001644345.1, GCA_001644365.1,
GCA_001644415.1, GCA_001644495.1, GCA_001644255.1, GCA_001644445.1, GCA_001644455.1,
GCA_001644245.1, GCA_015207155.1, GCA_015207075.1, GCA_015207195.1, GCA_015206905.1,
GCA_015207365.1, GCA_014132315.1, GCA_003003915.1, GCA_015233825.1, GCA_009915105.1,
GCA_018326565.1, GCA_018326585.1, GCA_018326665.1, GCA_018326685.1, GCA_018326705.1,
GCA_018326725.1, GCA_018326745.1, GCA_018326765.1, GCA_018326925.1, GCA_018327005.1,
GCA_018327065.1, GCA_018327085.1, GCA_018327145.1, GCA_018327205.1, GCA_018327385.1,
GCA_018327405.1, GCA_018327445.1, GCA_018327465.1, GCA_015223225.1, GCA_015234195.1,
GCA_009767105.1, GCA_015700665.1, GCA_015700695.1, GCA_010692825.1, GCA_014695345.1,
GCA_014695275.1, GCA_014695325.1, GCA_014695255.1, GCA_014695195.1, GCA_009900665.1,
GCA_009908265.2, GCA_018449595.1, GCA_018449715.1, GCA_014892285.1, GCA_014892275.1,
GCA_014892335.1, GCA_014892345.1, GCA_014892395.1, GCA_014892495.1, GCA_904424815.1,
GCA_015863215.1, GCA_015863195.1, GCA_015863185.1, GCA_014174405.1, GCA_014116815.1,
GCA_016123485.1, GCA_014384745.1, GCA_014384795.1, GCA_014384805.1, GCA_014384785.1,
GCA_014384885.1, GCA_014384905.1, GCA_014384765.1, GCA_014384895.1, GCA_014384705.1,
GCA_014385265.1, GCA_014384965.1, GCA_014384925.2, GCA_014384995.1, GCA_014385225.1,
GCA_014384985.1, GCA_014385005.1, GCA_014385025.1, GCA_014385135.2, GCA_014385165.1,

GCA_014385105.1, GCA_014385095.1, GCA_014385085.1, GCA_014385195.1, GCA_014306095.1, GCA_015472025.1, GCA_014779695.1, GCA_014858005.1, GCA_014841525.1, GCA_014841595.1, GCA_014841765.1, GCA_014841835.1, GCA_014841995.1, GCA_014842315.1, GCA_014857905.1, GCA_015206945.1, GCA_015206935.1, GCA_015207035.1, GCA_016587355.1, GCA_016587455.1, GCA_014982975.1, GCA_014982785.1, GCA_014982745.1, GCA_014982715.1, GCA_014983025.1, GCA_015476615.1, GCA_015546655.1, GCA_018524345.1, GCA_015689585.1, GCA_015689535.1, GCA_015689545.1, GCA_015689575.1, GCA_015689655.1, GCA_015689635.1, GCA_015689685.1, GCA_015689645.1, GCA_015689705.1, GCA_015689745.1, GCA_015689735.1, GCA_015689785.1, GCA_015689775.1, GCA_015689805.1, GCA_016464625.1, GCA_016522115.1, GCA_016458225.1, GCA_016595455.1, GCA_016595575.1, GCA_016811075.1, GCA_016892645.1, GCA_016892445.1, GCA_016918765.1, GCA_018332375.1, GCA_017948655.1, GCA_018348555.1, GCA_018348615.1, GCA_018348635.1, GCA_018682635.1, GCA_017114885.1, GCA_017942165.1, GCA_018145715.1, GCA_018122655.1, GCA_018223375.1, GCA_018223435.1, GCA_018362825.1, GCA_018362815.1, GCA_018362855.1, GCA_018413565.1, GCA_019083985.1, GCA_018982925.1, GCA_019008365.1, GCA_018883545.1, GCA_018919725.1, GCA_018919645.1, GCA_018919585.1, GCA_018919625.1, GCA_018919565.1, GCA_019084065.1, GCA_019061145.1, GCA_019084025.1, GCA_019132875.1, GCA_019166125.1, GCA_019218305.1, GCA_019218315.1, GCA_019218365.1, GCA_019218375.1, GCA_019218415.1, GCA_019218445.1, GCA_015550775.1, GCA_015551295.1, GCA_015553565.1, GCA_015554585.1, GCA_015557515.1, GCA_015559035.1, GCA_015560015.1, GCA_015669665.1, GCA_018784685.1, GCA_018784725.1, GCA_015555245.1, GCA_015559165.1, GCA_015559785.1, GCA_015561015.1, GCA_015667115.1, GCA_000428245.1, GCA_011800315.1, GCA_015207585.1, GCA_013519935.1, GCA_003426325.1, GCA_003426525.1, GCA_003426555.1, GCA_003585675.1, GCA_002968105.1, GCA_002968095.1, GCA_003030805.1, GCA_014610835.1, GCA_014156375.1, GCA_004791555.1, GCA_018326505.1, GCA_018326525.1, GCA_018326545.1, GCA_018326625.1, GCA_018326645.1, GCA_018326785.1, GCA_018326805.1, GCA_018326825.1, GCA_018326845.1, GCA_018326865.1, GCA_018326885.1, GCA_018326905.1, GCA_018326945.1, GCA_018326965.1, GCA_018326985.1, GCA_018327025.1, GCA_018327045.1, GCA_018327105.1, GCA_018327125.1, GCA_018327165.1, GCA_018327185.1, GCA_018327225.1, GCA_018327245.1, GCA_018327265.1, GCA_018327285.1, GCA_018327305.1, GCA_018327325.1, GCA_018327345.1, GCA_018327365.1, GCA_018327425.1, GCA_018784815.1, GCA_014873415.1, GCA_014873965.1, GCA_016467405.1, GCA_015700715.1, GCA_016617495.1, GCA_016617515.1, GCA_014529625.1, GCA_014529675.1, GCA_014529665.1, GCA_015354435.1, GCA_015354475.1, GCA_017898095.1, GCA_014837105.1, GCA_014837035.1, GCA_014836765.1, GCA_015666175.1, GCA_018598225.1, GCA_018598175.1, GCA_018598585.1, GCA_018598185.1, GCA_015905305.1, GCA_015905225.1, GCA_015905245.1, GCA_015550005.1, GCA_015669005.1, GCA_016649355.1, GCA_016649365.1, GCA_016649425.1, GCA_017810195.1, GCA_016521575.1, GCA_016469015.1, GCA_016632365.1, GCA_016918935.1, GCA_019140855.1, GCA_019148575.1, GCA_017948555.1, GCA_018128545.1, GCA_017592665.1, GCA_018223385.1, GCA_018223415.1, GCA_018223365.1, GCA_018704065.1, GCA_018729455.1, GCA_019042235.1, GCA_019042245.1, GCA_019042275.1

Supplemental Table S3. Functional annotation and protein identification benchmark set comprising 198 selected metagenome-assembled genomes from GenBanks

GenBank accession
GCA_002297765.1, GCA_002298295.1, GCA_002298975.1, GCA_002298225.1, GCA_002297475.1,
GCA_002295925.1, GCA_002293145.1, GCA_002314135.1, GCA_002311805.1, GCA_002308695.1,
GCA_002307725.1, GCA_002306875.1, GCA_002306465.1, GCA_002306155.1, GCA_002306335.1,
GCA_002305805.1, GCA_002325565.1, GCA_002325245.1, GCA_002325045.1, GCA_002322275.1,
GCA_002321895.1, GCA_002320175.1, GCA_002320085.1, GCA_002320055.1, GCA_002320005.1,
GCA_002337405.1, GCA_002337285.1, GCA_002337145.1, GCA_002337125.1, GCA_002336245.1,
GCA_002335085.1, GCA_002335425.1, GCA_002333005.1, GCA_002332985.1, GCA_002331575.1,
GCA_002331385.1, GCA_002329025.1, GCA_002328995.1, GCA_002328335.1, GCA_002328165.1,
GCA_002347645.1, GCA_002345215.1, GCA_002345185.1, GCA_002345145.1, GCA_002344965.1,
GCA_002344785.1, GCA_002344345.1, GCA_002343985.1, GCA_002342295.1, GCA_002341725.1,
GCA_002341585.1, GCA_002341545.1, GCA_002341485.1, GCA_002340805.1, GCA_002340585.1,
GCA_002340255.1, GCA_002359515.1, GCA_002359395.1, GCA_002359265.1, GCA_002359215.1,
GCA_002359185.1, GCA_002354635.1, GCA_002354605.1, GCA_002353655.1, GCA_002352555.1,
GCA_002352105.1, GCA_002348315.1, GCA_002346225.1, GCA_002367555.1, GCA_002366545.1,
GCA_002365175.1, GCA_002364745.1, GCA_002364215.1, GCA_002364085.1, GCA_002366485.1,
GCA_002378905.1, GCA_002378005.1, GCA_002376875.1, GCA_002376725.1, GCA_002376625.1,
GCA_002376045.1, GCA_002375365.1, GCA_002375495.1, GCA_002375465.1, GCA_002373505.1,
GCA_002373475.1, GCA_002371595.1, GCA_002372835.1, GCA_002369135.1, GCA_002393145.1,
GCA_002392595.1, GCA_002392045.1, GCA_002391865.1, GCA_002385275.1, GCA_002385225.1,
GCA_002385025.1, GCA_002384665.1, GCA_002384935.1, GCA_002382325.1, GCA_002382705.1,
GCA_002381615.1, GCA_002380645.1, GCA_002381125.1, GCA_002395615.1, GCA_002394385.1,
GCA_002389905.1, GCA_002390845.1, GCA_002400415.1, GCA_002389085.1, GCA_002389665.1,
GCA_002388685.1, GCA_002388665.1, GCA_002387375.1, GCA_002386415.1, GCA_002406015.1,
GCA_002405475.1, GCA_002405165.1, GCA_002403705.1, GCA_002403465.1, GCA_002403435.1,
GCA_002403335.1, GCA_002402585.1, GCA_002399785.1, GCA_002399025.1, GCA_002397905.1,
GCA_002415215.1, GCA_002414885.1, GCA_002415705.1, GCA_002415665.1, GCA_002414525.1,
GCA_002415565.1, GCA_002414445.1, GCA_002414385.1, GCA_002415385.1, GCA_002413005.1,
GCA_002412965.1, GCA_002410345.1, GCA_002410145.1, GCA_002424395.1, GCA_002427365.1,
GCA_002424235.1, GCA_002426725.1, GCA_002425805.1, GCA_002425185.1, GCA_002421065.1,
GCA_002420425.1, GCA_002420525.1, GCA_002420305.1, GCA_002419775.1, GCA_002419535.1,
GCA_002418285.1, GCA_002418245.1, GCA_002418095.1, GCA_002432845.1, GCA_002431665.1,
GCA_002431625.1, GCA_002430265.1, GCA_002427895.1, GCA_002427815.1, GCA_002427755.1,
GCA_002428995.1, GCA_002435785.1, GCA_002440635.1, GCA_002439045.1, GCA_002438025.1,
GCA_002437725.1, GCA_002439815.1, GCA_002436875.1, GCA_002433365.1, GCA_002456035.1,

GCA_002455715.1, GCA_002454815.1, GCA_002453945.1, GCA_002452095.1, GCA_002449395.1, GCA_002450875.1, GCA_002449845.1, GCA_002492145.1, GCA_002471845.1, GCA_002471675.1, GCA_002471575.1, GCA_002471335.1, GCA_002471125.1, GCA_002470515.1, GCA_002476985.1, GCA_002479255.1, GCA_002477825.1, GCA_002484125.1, GCA_002484005.1, GCA_002483535.1, GCA_002482645.1, GCA_002482605.1, GCA_002482265.1, GCA_002480685.1, GCA_002480575.1, GCA_002500565.1, GCA_002501065.1, GCA_002499995.1

Supplemental Notes

Supplemental Notes S1

During the SQLite database initialization procedure, UPSs, IPSs and PSCs records are created, importing unique protein sequences from UniRef100 and UniParc and protein cluster seed sequences from UniRef90 [3]. Subsequently, these database records are refined with annotations from additional external databases. Protein products and gene symbols are extracted from RefSeq non-redundant protein records [4], UniProt/SwissProt [3], AMRFinderPlus [5] or ISfinder [6]. Furthermore, annotations are enriched with additional information like EC numbers, COG functional categories [7] and GO [8] terms. All annotations are conducted and supersede each other in the outlined order according to the specificity of annotation sources.

1. RefSeq non-redundant proteins: UPSs are annotated with identifiers of RefSeq non-redundant protein records which are merged via PSHDs. IPSs or related PSCs are annotated with gene symbols.
2. NCBI COG: PSCs are annotated with COG function and category records via a large-scale homology search against cluster representative sequences of the COG [7] database using Diamond [9] applying a mutual coverage threshold 0.80 and a sequence identity threshold of 0.90. PSCs without a gene symbol or protein product different from *hypothetical protein* are annotated accordingly.
3. UniProt/SwissProt: IPSs and PSCs are annotated with gene symbols, protein products, EC numbers and GO terms from related SwissProt [3] records identified via PSHDs.
4. NCBI AMRFinderPlus: IPSs are annotated with gene symbols and protein products via PSHDs. PSCs are annotated with gene symbols and protein products by HMMER [10] hits against AMRFinderPlus HMMs.
5. ISfinder: IPSs and PSCs are annotated with gene symbols and protein products via large-scale homology searches against transposase protein sequences using Diamond [9] with a mutual coverage threshold of 0.99 and 0.80 and a sequence identity threshold of 0.98 and 0.90 for IPSs and PSCs, respectively.

Supplemental Notes S2

To provide high-quality annotation for certain genes of special interest that might not be able to sufficiently annotate via protein sequence hash digests or protein cluster sequences, Bakta takes advantage of a protein sequence-based expert annotation system integrating high-quality annotation sources from external databases. Therefore, protein sequences, gene symbols, protein products, query and subject coverage thresholds, sequence identity thresholds and priority ranks are stored for protein sequences from VFDB [11] and NCBI BlastRules [4]. To give precedence on more precise annotations in case of multiple annotations, these different sources are given a rank taking into account proprietary priority levels:

NCBI BlastRules BlastRuleEquivalog: 70
VFDB core sequences: 75
NCBI BlastRules BlastRuleException: 80
NCBI BlastRules BlastRuleIS: 90
NCBI BlastRules BlastRuleExact: 99

Supplemental Notes S3

Prokka, DFAST and Bakta were executed with the following command line options:

Prokka

```
prokka --outdir . --force --prefix <accession> --genus <genus>
--species <species> --strain <strain> --gcode 11 --usegenus --cpus 8
--rfam <input>
```

DFAST

```
dfast --out output --organism "<genus> <species>" --strain <strain>
--use_original_name t --sort_sequence f --cpu 8 --genome <input>
```

Bakta

```
bakta --db <baktadb> --prefix <prefix> --genus <genus> --species
<species> --strain <strain> --keep-contig-headers --threads 8
--complete <input>
```

Supplemental Notes S4

The backend of our Bakta web application is based on a scalable REST-API implemented in Go. All input data is isolated on a per-job level, by the provision of dedicated cloud based object storage endpoints, *i.e.* buckets, for each annotation job. Actual annotations are conducted by processes of the Bakta command line version distributed on a scalable Kubernetes cluster. Currently, this Kubernetes cluster is hosted and run within a cloud computing infrastructure of the de.NBI consortium. Hence, required hardware resources can be seamlessly scaled to current demands. Besides accepting jobs from our web application, this public API can also be used independently as an annotation microservice, *e.g.* for the integration into larger web-based analysis pipelines. A comprehensive documentation of this API is provided at https://bakta.readthedocs.io.

Supplemental References

1. Parks DH, Rinke C, Chuvochina M, Chaumeil P-A, Woodcroft BJ, Evans PN, et al. Recovery of nearly 8,000 metagenome-assembled genomes substantially expands the tree of life. Nat Microbiol [Internet]. 2017 Nov;2(11):1533–42. Available from: http://dx.doi.org/10.1038/s41564-017-0012-7

2. Parks DH, Imelfort M, Skennerton CT, Hugenholtz P, Tyson GW. CheckM: assessing the quality of microbial genomes recovered from. Cold Spring Harbor Laboratory Press Method [Internet]. 2015;1–31. Available from: http://dx.doi.org/10.1101/gr.186072.114

3. UniProt Consortium. UniProt: the universal protein knowledgebase in 2021. Nucleic Acids Res [Internet]. 2021 Jan 8;49(D1):D480–9. Available from: http://dx.doi.org/10.1093/nar/gkaa1100

4. Li W, O'Neill KR, Haft DH, DiCuccio M, Chetvernin V, Badretdin A, et al. RefSeq: expanding the Prokaryotic Genome Annotation Pipeline reach with protein family model curation. Nucleic Acids Res [Internet]. 2021 Jan 8;49(D1):D1020–8. Available from: http://dx.doi.org/10.1093/nar/gkaa1105

5. Feldgarden M, Brover V, Gonzalez-Escalona N, Frye JG, Haendiges J, Haft DH, et al. AMRFinderPlus and the Reference Gene Catalog facilitate examination of the genomic links among antimicrobial resistance, stress response, and virulence. Sci Rep [Internet]. 2021 Jun 16;11(1):12728. Available from: http://dx.doi.org/10.1038/s41598-021-91456-0

6. Siguier P, Perochon J, Lestrade L, Mahillon J, Chandler M. ISfinder: the reference centre for bacterial insertion sequences. Nucleic Acids Res [Internet]. 2006 Jan 1;34(Database issue):D32–6. Available from: http://dx.doi.org/10.1093/nar/gkj014

7. Galperin MY, Wolf YI, Makarova KS, Vera Alvarez R, Landsman D, Koonin EV. COG database update: focus on microbial diversity, model organisms, and widespread pathogens. Nucleic Acids Res [Internet]. 2021 Jan 8 [cited 2020 Nov 10];49(D1):D274–81. Available from: http://dx.doi.org/10.1093/nar/gkaa1018

8. Gene Ontology Consortium. The Gene Ontology resource: enriching a GOld mine. Nucleic Acids Res [Internet]. 2021 Jan 8;49(D1):D325–34. Available from: http://dx.doi.org/10.1093/nar/gkaa1113

9. Buchfink B, Xie C, Huson DH. Fast and sensitive protein alignment using DIAMOND. Nat Methods [Internet]. 2015 Jan;12(1):59–60. Available from: http://dx.doi.org/10.1038/nmeth.3176

10. Eddy SR. Accelerated Profile HMM Searches. PLoS Comput Biol [Internet]. 2011 Oct;7(10):e1002195. Available from: http://dx.doi.org/10.1371/journal.pcbi.1002195

11. Liu B, Zheng D, Jin Q, Chen L, Yang J. VFDB 2019: a comparative pathogenomic platform with an interactive web interface. Nucleic Acids Res [Internet]. 2019 Jan

8;47(D1):D687–92. Available from: http://dx.doi.org/10.1093/nar/gky1080
